# Neuropsychiatric Circuitry and Receptor Dysregulation in the Pathogenesis of Bruxism

**DOI:** 10.1192/j.eurpsy.2025.2116

**Published:** 2025-08-26

**Authors:** D. Patel, M. Murugappan, B. Carr

**Affiliations:** 1 Medicine, Nova Southeastern University, Davie; 2 Psychiatry, University of Florida, Gainesville, United States

## Abstract

**Introduction:**

Bruxism, characterized by the grinding and clenching of teeth, is often associated with psychiatric disorders such as anxiety and stress. Bruxism not only results in significant dental pathology but can also contribute to underlying neurophysiological disturbances.

**Objectives:**

To elucidate the relationship between bruxism and psychiatric medication by focusing on the neurophysiological mechanisms involved and the resultant dental pathologies.

**Methods:**

A comprehensive literature review was conducted using databases such as PubMed, PsycINFO, and Google Scholar, focusing on studies from the last decade that investigate the association between bruxism, psychiatric medications, and neurophysiological factors. The review included clinical studies, neuroimaging research, and behavioral analyses.

**Results:**

The findings indicate a strong association between bruxism and the use of psychiatric medications, particularly antidepressants and antipsychotics. Neurophysiological studies reveal dysregulation in neurotransmitter systems, notably dopamine and serotonin, which play critical roles in both bruxism and the effects of psychiatric medications. This dysregulation affects motor control circuits and stress response pathways in the central nervous system, leading to involuntary teeth grinding and clenching.

**Table 1: Neurophysiological Mechanisms**

**Table:** 

Mechanism	Description
Dopamine Dysregulation	Inhibition of dopaminergic neurons leads to dysregulation of motor control and contributes to spontaneous movement of jaw muscles.
Serotonin Imbalance	Excess serotonin enhances excitatory neurotransmission and disrupts dopaminergic pathways, contributing to increased anxiety and masseter muscle hyperactivity.
Autonomic Nervous System	Hyperactivity in the sympathetic branch, driven by chronic stress, leads to increased arousal and muscle tone causing bruxism.

**Table 2: Dental Pathologies Resulting from Bruxism**

**Table:** 

Pathology	Description
Tooth Wear	Enamel erosion due to repetitive grinding, leading to dentin exposure.
Fractures	Microfractures in teeth from constant pressure, progressing to severe cracks.
TMJ Disorders (TMJD)	Chronic bruxism contributes to TMJD, characterized by pain and joint dysfunction.
Periodontal Damage	Excessive force on teeth exacerbates periodontal issues, leading to gum recession.

**Conclusions:**

Bruxism is both a symptom and a potential side effect of various psychiatric medications, rooted in neurophysiological disturbances. The interplay between dysregulated neurotransmitter systems, psychiatric medications, and resultant dental pathologies highlights the need for integrated dental and psychiatric care. Effective management of bruxism through targeted dental interventions and tailored psychiatric treatments can significantly improve both dental health and psychiatric well-being.

**Disclosure of Interest:**

None Declared

